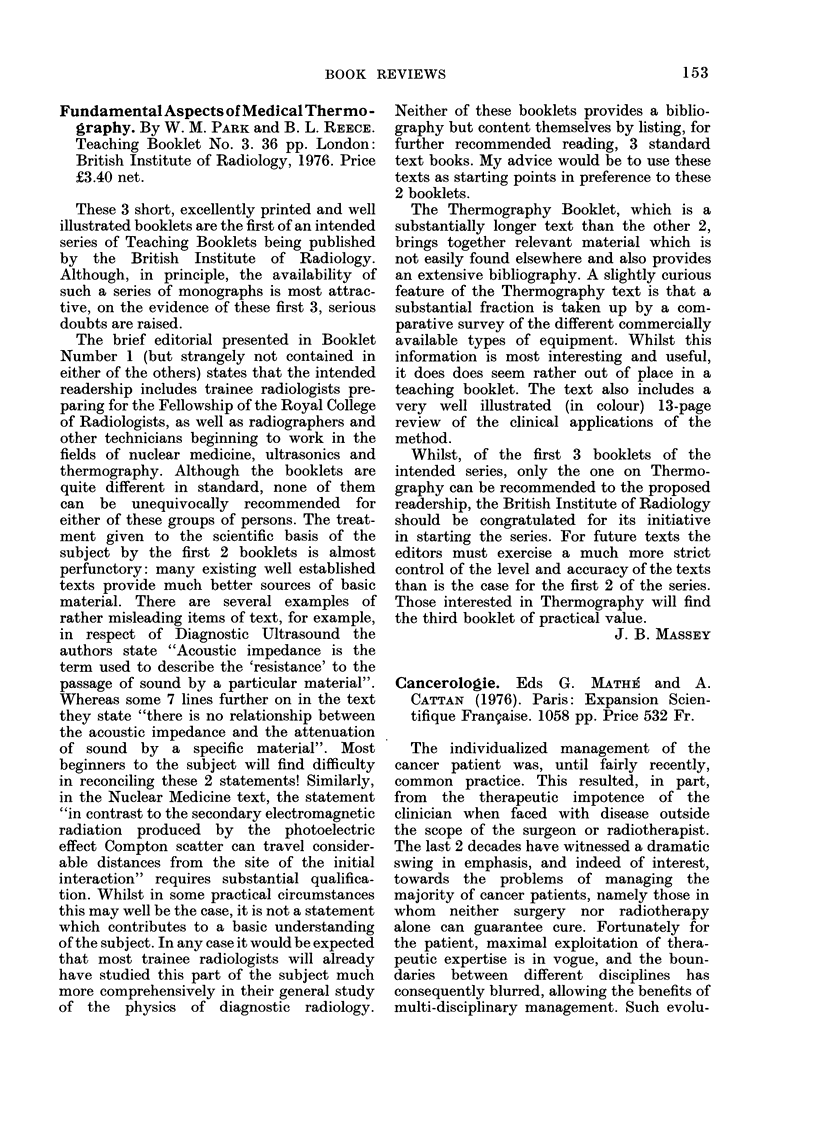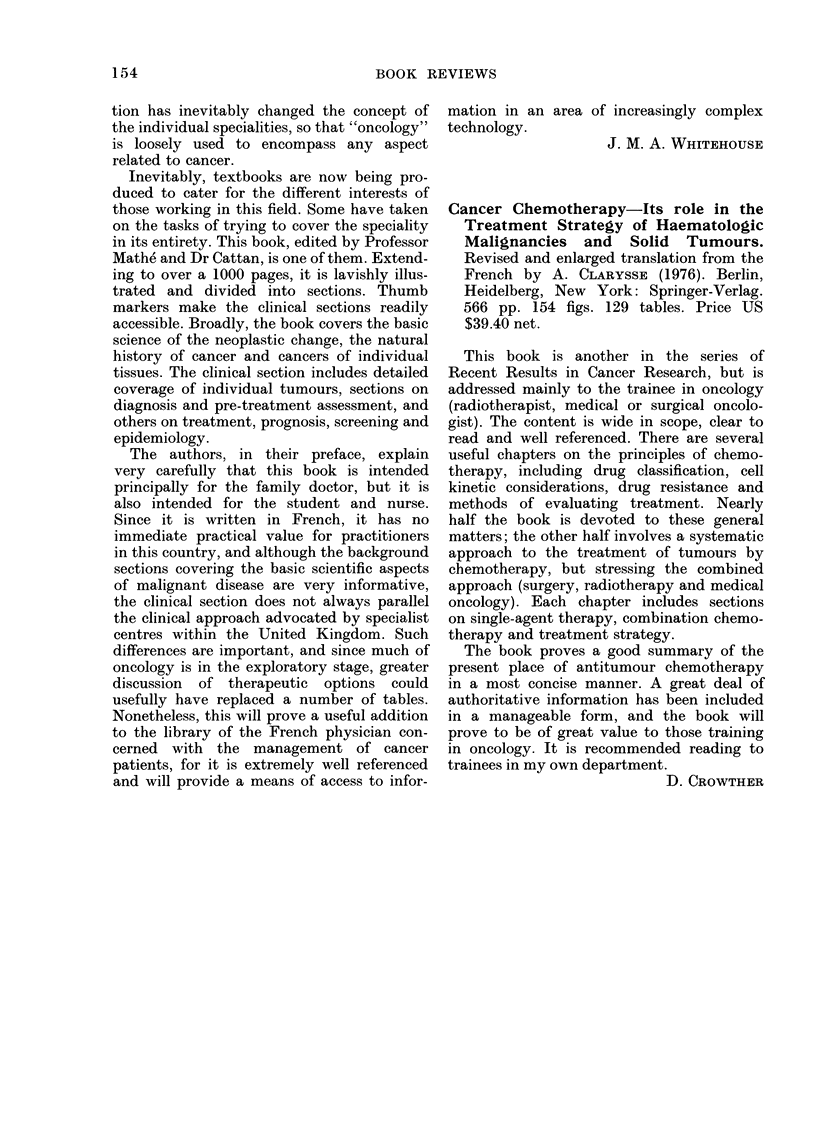# Cancerologie

**Published:** 1977-07

**Authors:** J. M. A. Whitehouse


**Cancerologie.** Eds G. MATHÉ and A. CATTAN (1976). Paris: Expansion Scientifique Française. 1058 pp. Price 532 Fr.

The individualized management of the cancer patient was, until fairly recently, common practice. This resulted, in part, from the therapeutic impotence of the clinician when faced with disease outside the scope of the surgeon or radiotherapist. The last 2 decades have witnessed a dramatic swing in emphasis, and indeed of interest, towards the problems of managing the majority of cancer patients, namely those in whom neither surgery nor radiotherapy alone can guarantee cure. Fortunately for the patient, maximal exploitation of therapeutic expertise is in vogue, and the boundaries between different disciplines has consequently blurred, allowing the benefits of multi-disciplinary management. Such evolution has inevitably changed the concept of the individual specialities, so that "oncology" is loosely used to encompass any aspect related to cancer.

Inevitably, textbooks are now being produced to cater for the different interests of those working in this field. Some have taken on the tasks of trying to cover the speciality in its entirety. This book, edited by Professor Mathé and Dr Cattan, is one of them. Extending to over a 1000 pages, it is lavishly illustrated and divided into sections. Thumb markers make the clinical sections readily accessible. Broadly, the book covers the basic science of the neoplastic change, the natural history of cancer and cancers of individual tissues. The clinical section includes detailed coverage of individual tumours, sections on diagnosis and pre-treatment assessment, and others on treatment, prognosis, screening and epidemiology.

The authors, in their preface, explain very carefully that this book is intended principally for the family doctor, but it is also intended for the student and nurse. Since it is written in French, it has no immediate practical value for practitioners in this country, and although the background sections covering the basic scientific aspects of malignant disease are very informative, the clinical section does not always parallel the clinical approach advocated by specialist centres within the United Kingdom. Such differences are important, and since much of oncology is in the exploratory stage, greater discussion of therapeutic options could usefully have replaced a number of tables. Nonetheless, this will prove a useful addition to the library of the French physician concerned with the management of cancer patients, for it is extremely well referenced and will provide a means of access to information in an area of increasingly complex technology.

J. M. A. Whitehouse